# Dual chamber pacemaker implantation in woman with twin pregnancy and Chagas cardiomyopathy guided by 2D transthoracic echocardiography

**DOI:** 10.1016/j.ipej.2021.10.001

**Published:** 2021-10-08

**Authors:** Paul M. Preza, Mauricio Guerra, Ladys R. Cárdenas, Victoria C. Armas

**Affiliations:** Hospital Nacional Arzobispo Loayza, Av. Alfonso Ugarte 848, Lima, PC 15082, Peru

**Keywords:** Chagas cardiomyopathy, Twin pregnancy, Transthoracic echocardiography, Sick sinus syndrome, Artificial pacemaker

## Abstract

We present a case of 36-year-old woman with twin pregnancy, Chagas cardiomyopathy and history of multiple episodes of dizziness and syncope. The patient's Holter study revealed sinus pauses of up to 5.3 seconds, frequent premature ventricular contractions (PVC) and some episodes of non-sustained ventricular tachycardia at 110 bpm. To avoid teratogenic radiation, dual chamber pacemaker implantation was performed guided by transthoracic echocardiography. The patient was treated with metoprolol succinate 100 mg once a day to reduce PVC and nonsustained ventricular tachycardia. During follow up, the patient reported complete resolution of syncope and dizziness. She went on to have a normal delivery without complications. PCRs for Chagas in both twins were negative.

A 36-year-old woman originally from the indigenous community of Andoas in the jungle of Perú was referred to our hospital in Lima. She arrived with a 2-years history of fatigue, dizziness and one year of recurrent syncope. At the time of admission, she was 16 weeks pregnant with a viable twin pregnancy.

The Holter study revealed sinus pauses of up to 5.3 seconds, frequent premature ventricular contractions (PVC) and some episodes of non-sustained ventricular tachycardia at 110 bpm (Fig. 1A) Serological tests for Chagas disease were positive.

**Fig. 1:**
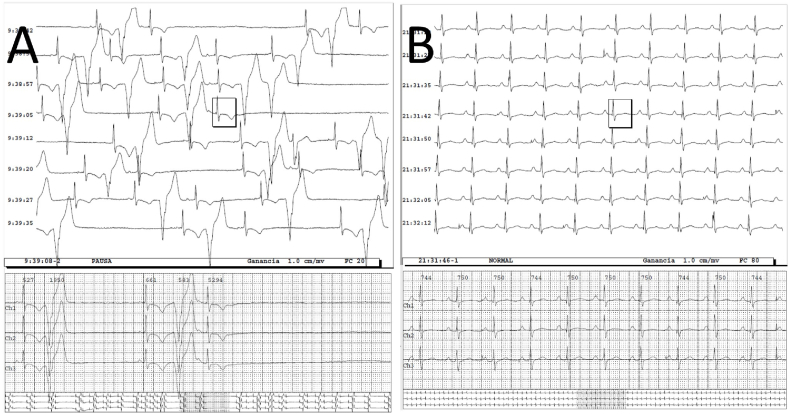
Figure A shows the pre implantation Holter with frequent PVC and several pauses including one of 5.29s. Figure B shows the post implantation Holter showing pacemaker rhythm with atrial pacing and ventricular sensing.

Decision was made to proceed with implantation of a dual chamber permanent pacemaker guided by transthoracic echocardiography. The implantation technique is described below.

Two-dimensional transthoracic echocardiography was performed with a Phillips i33 machine. Right ventricle lead (Biotronik Safio S60) was implanted using the technique previously described by Güldal [1]. Thereafter, the atrial lead (Biotronik Safio S53), with a J stylet inside, was introduced through the left subclavian vein with the distal curve and the point of its tip towards the anterior region of the chest (Fig. 2C). After applying clockwise and counter-clockwise torque alternatively on the lead, the tip of the atrial lead was placed in the right atrial appendage. Lead positioning and stability were also confirmed by echocardiography through a subxiphoid window under the following approaches: in the bicaval view, it was possible to visualize the entire route of the atrial lead within the right atrium (Fig. 2A, B, C and online video 1) and in the four-chamber view, it was possible to confirm the so-called windshield wiper appearance of the atrial lead motion (online video 2). After that, the retractable screw was deployed for active fixation, and the capture thresholds were verified at 0.7mV/0.4s both in the atrium and in the ventricle. The procedure was completed according to the usual technique without any complication.

**Fig. 2:**
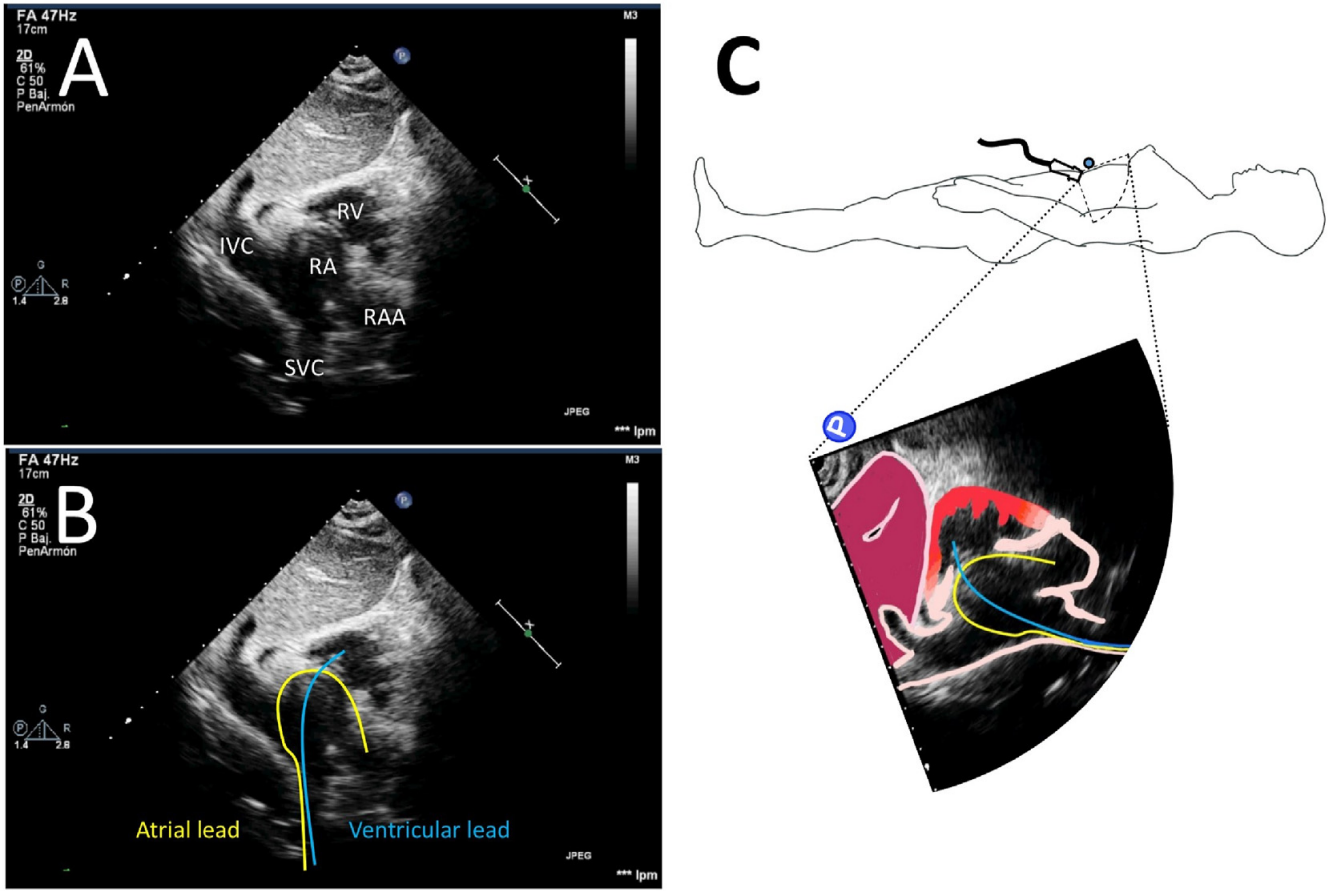
The figure A shows a subxiphoid window of the 2D transthoracic echocardiography taken intraprocedural. Figure B is for enhance the recognition of the leads in this view. Figure C shows a graphical representation of the position of the echocardiography probe and beam. Notice how the curve and tip of the atrial lead point toward the front of the patient. RV right ventricle, IVC inferior vena cava, RA right atrium, RAA right atrial appendage, SVC superior vena cava.

The patient was treated with metoprolol succinate 100 mg once a day to reduce ventricular arrhythmias. Dual chamber pacemaker (Biotronik Etrinsa 6 DR-T) was programed in DDDR mode with a lower rate limit of 70 ppm to improve the cardiac output and to avoid R on T episodes related to the frequent premature ventricular contractions. The post implantation Holter study showed reduction of ventricular tachycardia episodes compared to the previous study. The patient remained paced in the atrium nearly 100% (Fig. 1B).

During follow up, the patient reported complete resolution of syncope and dizziness stopped. She went on to have a normal delivery without complications. PCRs for Chagas in both twins were negative.

As far as we're concerned, this is the first description of dual chamber permanent pacemaker implantation guided by transthoracic echocardiography.

## Credit author statement

Paul M Preza: Clinical management and follow up, original draft preparation.

Mauricio Guerra: Clinical management, Writing, Reviewing and Editing.

Ladys R. Cárdenas: Echocardiography Images.

Victoria C Armas: Supervision.

## Funding support

None.

“All authors have read and approved the manuscript.”

## Declaration of competing interest

The authors have no conflicts to disclose.
